# Does Renal Denervation a Reasonable Treatment Option in Hemodialysis-Dependent Patient with Resistant Hypertension? A Narrative Review

**DOI:** 10.1007/s11906-023-01264-2

**Published:** 2023-09-06

**Authors:** Alberto Mazza, Fabio Dell’Avvocata, Gioia Torin, Francesca Bulighin, Yuri Battaglia, Fulvio Fiorini

**Affiliations:** 1 ESH Excellence Hpertension Centre and Dept. of Internal Medicine, Santa Maria della Misericordia General Hospital, AULSS 5 Polesana, Rovigo, Italy; 2 Cardiovascular Diagnosis and Endoluminal Interventions Unit, Santa Maria della Misericordia General Hospital, AULSS 5 Polesana, Rovigo, Italy; 3 Department of Medicine, University of Verona, 37129 Verona, Italy; 4 Nephrology and Dialysis Unit, Pederzoli Hospital, Via Monte Baldo, 24, 37019 Peschiera del Garda, Italy; 5 Nephrology, Dialysis and Dietology Unit, Santa Maria della Misericordia General Hospital, AULSS 5 Polesana, Rovigo, Italy

**Keywords:** Nephrectomy, Renal failure, Ultrasound, Renin-angiotensin system inhibitors, End-stage kidney disease, Radiofrequency ablation, Sympathetic overactivity, Blood pressure

## Abstract

**Purpose of Review:**

This narrative review aims to assess the pathophysiology, diagnosis, and treatment of resistant hypertension (RH) in end-stage kidney disease (ESKD) patients on dialysis, with a specific focus on the effect of renal denervation (RDN) on short-term and long-term blood pressure (BP) control. Additionally, we share our experience with the use of RDN in an amyloidotic patient undergoing hemodialysis with RH.

**Recent Findings:**

High BP, an important modifiable cardiovascular risk factor, is often observed in patients in ESKD, despite the administration of multiple antihypertensive medications. However, in clinical practice, it remains challenging to identify RH patients on dialysis treatment because of the absence of specific definition for RH in this context. Moreover, the use of invasive approaches, such as RDN, to treat RH is limited by the exclusion of patients with reduced renal function (eGFR < 45 mL/min/1.73 m3) in the clinical trials. Nevertheless, recent studies have reported encouraging results regarding the effectiveness of RDN in stage 3 and 4 chronic kidney disease (CKD) and ESKD patients on dialysis, with reductions in BP of nearly up to 10 mmhg.

**Summary:**

Although multiple underlying pathophysiological mechanisms contribute to RH, the overactivation of the sympathetic nervous system in ESKD patients on dialysis plays a crucial role. The diagnosis of RH requires both confirmation of adherence to antihypertensive therapy and the presence of uncontrolled BP values by ambulatory BP monitoring or home BP monitoring. Treatment involves a combination of nonpharmacological approaches (such as dry weight reduction, sodium restriction, dialysate sodium concentration reduction, and exercise) and pharmacological treatments. A promising approach for managing of RH is based on catheter-based RDN, through radiofrequency, ultrasound, or alcohol infusion, directly targeting on sympathetic overactivity.

## Introduction

Resistant hypertension (RH) is frequently observed in patients with chronic kidney disease (CKD). Despite anti-hypertensive treatment, it can lead to the progression of kidney function decline due to the sustained elevation in blood pressure (BP) level [1].

Severe RH in patients undergoing dialysis (HD) has traditionally been an indication for bilateral nephrectomy [2]. However, it is infrequently carried out since the clinical benefits in improving BP values usually do not balance the high peri-operative morbidity risks. Nonetheless, bilateral nephrectomy may be considered in rare cases of non-compliant patients with life-threatening hypertension that cannot be controlled with any other intervention [3, 4].

In recent years, observations on bilateral native nephrectomy as antihypertensive treatment have provided the rationale for catheter-based renal denervation in CKD patients with true RH. Indeed, surgical ablation ameliorates sympathetic overactivity and prevents both hypertension (HT) and the progression of renal disease in experimental models. In this scenario, a percutaneous approach with bilateral renal sympathetic denervation using a radiofrequency ablation procedure has shown promising results as a new available therapeutic strategy in this condition [5].

With this background in mind, our aim is to provide a comprehensive review of the pathophysiology, diagnosis, and treatment of RH in patients with end-stage kidney disease (ESKD) undergoing dialysis. Specifically, we focus on examining the impact of renal denervation (RDN) on short-term and long-term BP control. Furthermore, we present our experience with the use of RDN in an amyloidotic patient receiving maintenance hemodialysis with RH.

## Search Strategy and Selection Criteria

We conducted a comprehensive search of the PubMed, Scopus, Google Scholar, and Web of Science databases for articles published from their inception up to April 30, 2023. We used the following search terms: “resistant hypertension,” “renal denervation,” “end-stage kidney disease,” “end-stage renal disease,” “kidney failure,” “renal replacement therapy,” “dialysis,” and “hemodialysis.” We primarily included articles published from January 1, 2013, to April 30, 2023, in the English language. However, we did not exclude relevant and highly referenced older publications.

## Resistant Hypertension

### Diagnosis and Definition

According to the 2017 American Heart Association and American College of Cardiology (AHA/ACC) hypertension guidelines [6], the 2018 European Societies of Cardiology and European Society of Hypertension (ESC/ESH) hypertension guidelines [7], the 2020 Hypertension Canada [8], and the 2023 European Society of Hypertension (ESH) hypertension guidelines endorsed by European Renal Association (ERA) and the International Society of Hypertension (ISH) [9], the diagnosis of RH is the same for patient with or without CKD or undergoing dialytic treatment. Specifically, RH is defined as having BP above the systolic BP and/or diastolic BP above targets, despite the concurrent use of three medications belonging to different antihypertensive classes, including a diuretic if tolerated. The maximum recommended or maximally tolerated doses of all antihypertensive drugs should be prescribed. In addition, patients who require four or more medications to control their BP are also considered to have RH. Although a threshold of 140/90 mmhg has traditionally been set for diagnosis of RH [10], the 2017 AHA/CC guidelines have reduced the BP goal at 130/80 mmhg [11].

True RH can be distinguished from pseudo-resistance if BP levels are above goal when measured in the dialysis using proper technique and confirmed with out-of-dialysis measurements, such as 24-h ambulatory BP monitoring (ABPM-24 h) or home BP monitoring, while excluding non-adherence to antihypertensive therapy [12].

However, the diagnostic accuracy of BP measurements, typically taken pre or post dialysis, is limited by several technical and patient-related factors such as incorrect BP evaluation and reading, volume fluctuations, anxious state, and the white-coat effect [13, 14]. Alternatively, ABPM-24 h, considered the gold standard for diagnosing hypertension in HD patients, offers some advantages, including BP nocturnal recording and a strict association with cardiovascular mortality, but also disadvantages, such as worsening sleep disorders and improved treatment burden [15]. Currently, home BP monitoring remains a safer and simpler methods to confirm a RH diagnosis in HD patients [16, 17].

### Pathophysiology

The primary cause of hypertension in dialysis patients is the sodium retention and volume expansion [18, 19]. When there is a volume overload, BP rise due to an increase in cardiac output and high systemic vascular resistance [20, 21]. Furthermore, a significant body of literature indicates that the correction of volume overload by removing excess sodium and reducing target dry weight can improve (BP) levels in approximately 60% of extracorporeal dialysis patients [22–24].

Other factors, such as endothelial dysfunction, activation of renin–angiotensin–aldosterone axis, and overactivity of sympathetic neural system, may also contribute to RH in dialysis patients [20]. Over the last few decades, there has been increased research interest in sympathetic nerve discharge. Native kidneys can send afferent nerve impulses to the central nervous system, leading to sympathetic overdrive [25]. Besides, sympathetic activity increases with CKD progression [26] and afferent sensory renal nerves, in response to intra-renal injury, can have an excitatory influence on central sympathetic outflow [27]. Therefore, renal sympathetic efferent and afferent nerves play a significant influence in the initiation, development, and maintenance of elevated systemic BP commonly detected in patients with end stage renal disease, often leading to RH [28].

### Autonomic and Reflex Effects of Dialysis Procedures

ESKD is characterized by significant modifications in the autonomic control of the cardiovascular system. These changes includes (1) heightened activity in the sympathetic nervous system that affects the cardiovascular system, (2) early onset of adrenergic abnormalities that are directly proportional to the severity of the renal dysfunction, (3) a decrease in the inhibitor influence of vagal nerve on sinus node, resulting in increased resting heart rates, (4) impaired modulation of both vagal and sympathetic cardiovascular effects by the arterial baroreceptors, (5) impaired control of sympathetic vasoconstrictor tone and renin release from the juxtaglomerular cells by cardiopulmonary receptor, (6) activation of chemoreflex, and (7) diminished sensitivity of the alpha adrenergic vascular receptors [29].

The extent to which hemodialysis can reverse and possibly normalize the altered autonomic profile of HD patients remains a matter of controversy. While few studies documented improvements in the parasympathetic tone of the heart rate after a single hemodialysis session [30], no significant change in autonomic dysfunction was observed in long-term HD patients, supporting the hypothesis that uremic neuropathy is irreversible and refractory to the hemodialysis treatment [31].

Moreover, these discrepancies among studies can also be attributed to the different dialytic modalities adopted. For example, nocturnal hemodialysis and short daily hemodialysis can trigger positive autonomic effects, enhancing arterial baroreflex sensitivity and reducing the sympathetic activity. On the other hand, peritoneal dialysis does not have a significant effect on autonomic dysfunction [32, 33].

Finally, volume overload may be another possible explanation of varying outcomes observed across different studies since central blood volume plays a crucial role in determining reflex responses [34].

## Management of Resistant Hypertension

### Non-pharmacologic Therapy

Despite the indication of antihypertensive drugs to achieve BP control, the benefits of non-pharmacological interventions for HD patients with RH should be considered.

Indeed, the effectiveness of anti-hypertensive therapy in hemodialysis patients relies on maintaining euvolemia [35, 36]. While various subjective and objective tools, such as questionaries, bio-impedance analysis, and ultrasound, are available to estimate dry weight, there is no agreement in the nephrology community on how to determine the euvolemic status of patients undergoing dialysis [37, 38]. Currently, computerized tomography (CT) remains the gold standard for assessing the different components of body, but its clinical use is limited by high radiation doses, cost, and impracticability [39, 40].

Among various non-pharmacological treatments for controlling the volume overload, salt restriction and dialysate sodium reduction are the most commonly used [41]. The available evidence supports a significant contribution of salt sensitivity to RH in HD patients [42]. Therefore, educating HD patients on a low salt diet is critical to achieving BP control while maintaining a simple medication regimen. A modest dietary sodium restriction can enhance the effects of antihypertensive medications such as angiotensin-converting enzyme inhibitors or angiotensin receptor blockers [43]. Although clinical trials are not available to definitively establish the benefits of sodium restriction in HD patients, observational studies indicated that reducing dietary sodium intake to a target of less than 50 mmol/day (approximately 3 g/day of salt) decreased systolic BP by up to 10 mmHg [37]. However, implementing a low sodium diet in clinical practice can be challenging due to various factors such as patient adherence and food preferences.

Similarly, high concentrations of sodium in dialysate are often used to reduce the risk of intradialytic hypotension, but on the other hand, they increase the sodium load and balance, interdialytic weight, thirst, and BP. An individualized strategy to optimize the dose of dialysate sodium should be assessed in clinical trials in HD patients with RH [44].

Furthermore, other studies, investigating the correlation of weight loss, assessed by BMI, with BP among HD patients, produced conflicting results. Although a link between higher BMI and higher BP has been demonstrated, a phenomenon referred to as the “paradoxical effect,” where an increase in BMI is inversely associated with BP levels, has been revealed [45].

Finally, another simple and efficient non-pharmacologic approach could be to prescribe intradialytic or interdialytic exercise in HD patients [44] who often maintain sedentary lifestyles [43, 46]. Small observational studies have shown encouraging results in reducing BP through intradialytic or interdialytic exercise even if a meta-analysis of 13 randomized controlled trials did not confirm these findings [47, 48]. Therefore, clinical trials should be conducted to evaluate the impact of physical exercise on BP in HD patients with resistant hypertension [49].

### Pharmacologic Therapy

Combination drug therapy is necessary to reach BP targets in patients with RH, including those on hemodialysis. However, the potential risks of polypharmacy should be also considered, given the high pill burden of HD patients. There is currently insufficient evidence to suggest the superiority of any class of antihypertensive medication, either alone or in combination, for HD population, regardless of individual preferences. Nevertheless, although some antihypertensive drugs may offer additional cardio-protective benefits beyond their direct BP-lowering effects, their possible side effects should also be taken into account. Ultimately, the choice of an anti-hypertensive drug should aim to balance reducing cardiovascular risk with minimizing adverse effects. In clinical practice, other relevant characteristics, such as the drug’s dialyzability and timing of administration in relation to hemodialysis, can also inform drug selection [44, 50].

#### Beta-Blockers

Some drugs belonging to the anti-hypertensive class of beta-blockers, including carvedilol and atenolol, have been studied in HD patients [51, 52]. The results of these trials showed not only anti-hypertensive effects but also a reduction in cardiovascular risk and composite cardiovascular outcomes when compared with a placebo or lisinopril. However, no data are available regarding the effect of b-blockers in combination with other anti-hypertensive drugs in HD patients with RH.

#### Renin-Angiotensin System Inhibitors

Renin–angiotensin–aldosterone system (RAAS) blockade, achieved through use of ACE inhibitors and angiotensin-II receptor blockers (ARBs), can reduce systolic BP, comparable to that achieved by calcium-channel blockers (CCBs) [53] in HD patients with hypertension. These drugs also exhibit cardio-protective properties, such as reducing left ventricular mass index, as demonstrated in a meta-analysis [54]. However, the positive effect of these drugs on cardiovascular risk is not clearly evident [55, 56]. Furthermore, hyperkaliemia is the main side effects associated with RAAS blockade [37] and current guidelines [57] recommended avoiding the dual blockade to reduce the risk of hyperkalemia and cardiovascular events.

#### Diuretics

The prescription of diuretics in HD patients with residual renal function for the treatment of the volume overload and improvement of left ventricular mass index and arterial stiffness remains a topic of debate with conflicting opinions. While loop diuretics have shown promising evidence in increasing urine output, no data support their efficacy in controlling BP in HD patients [58].

However, if tolerated, loop diuretics should always be a part of combination drug therapy in cases of resistant hypertension, and high doses should be used due to the tubular mechanism of action that relies on glomerular filtration [54]. In contrast, thiazide diuretics, such as bendroflumethiazide, or a thiazide-like diuretic, such as indapamide, should be discontinued in HD patients [17]. Additionally, although only one small trial has been conducted with mineralocorticoid receptor antagonists, such as spironolactone, in the HD population, showing no statically significant increase in hyperkalemia, these diuretics should be prescribed with high caution [59].

#### Calcium Channel Blockers (CCBs)

Although both dihydropyridine and non-dihydropyridine CCBs are useful in managing resistant hypertension, amlodipine, a dihydropyridine CCBs, has been extensively studied in HD patients. Therefore, it could be considered a first-line association therapy for RH for its effects not only in controlling BP but also in reducing the cardiovascular risk [60]. However, these promising results should be supported by further clinical trials [61].

### Adherence

In order to effectively control BP, the adherence to anti-hypertensive therapy is crucial in CKD patients, although CKD does not lead to a higher percentage of poor adherence to drugs compared to individuals without CKD [62].

Reduced drug adherence, which can further worsen when CKD patients start dialysis, is one of the most frequent factors contributing to RH [63]. However, it is worth to recognize that even if poor adherence is strongly linked to resistant hypertension, its significance differs significantly.

Studies examining the reasons behind non-compliance with anti-hypertensive treatment in HD patients emphasize the significance of effective communication and the perceived benefit of the therapies, regardless of the antihypertensive class [64].

In addition, other factors, including pill burden, drug interactions, and adverse effects, play a significant role in non-adherence to anti-hypertensive treatment [65, 66]. Therefore, antihypertensive regimens should be simplified whenever possible, considering the quantity, timing, and formulation of interventions. Continuity of care may also have a positive impact on patient outcome and, if feasible, efforts should be made to ensure that HD patients are able to see the same clinician at each visit. A systematic review has demonstrated that maintaining continuity in healthcare providers can improve outcomes for patients [67].

## Renal Denervation: Devices and Modalities

The two most extensively studied platforms for renal denervation (RDN) are radiofrequency RDN (rRDN) and ultrasound RDN (uRDN). In both procedures, the renal artery is accessed via the common femoral artery after selective renal angiography and placement of a 0.014″ guidewire in the renal arteries. It is worth highlighting that despite rRDN is designed to treat both the main and distal renal arteries while uRDN specifically the main renal arteries, the efficacy of both procedures is comparable [68•, 69].

### Radiofrequency Technology

rRDN is currently performed using the Symplicity Spyral™ RDN system (Medtronic Inc.), which utilizes radiofrequency waves emitted from four radiopaque electrodes located on the helical shaped tip of the Spyral™ catheter [70].

### Ultrasound Technology

uRDN is performed using the Paradise Renal Denervation System (ReCor Medical), which incorporates a balloon at its distal tip surrounding an ultrasound emitting core. The Paradise catheter is an over-the-wire system connected to a console which inflates the balloon and continuously infuses sterile water to cool the arterial wall before, during, and after the treatment [70].

### Others

In addition of the aforementioned technologies, ongoing clinical trials such as TARGET BP I [71] and TARGET BP OFF-MED [72] are investigating RDN using perivascular alcohol infusion in patients on and off antihypertensive medication, respectively.

## Autonomic Effects of Renal Nerve Ablation

In the first clinical trials investigating the efficacy and tolerability of percutaneous ablation of renal nerves in treatment of RH, renal dysfunction was listed as one of the exclusion criteria. Nevertheless, only a few studies have recently examined the effects of the RDN in patients with CKD stages III and IV, providing compelling evidence that RDN do not affect renal function [73].

Furthermore, studies with long-term follow-up indicate RDN may even slow the progression of CKD and, in some cases, lead to improvement in albumin and protein urinary excretion [70, 74].

However, it is still unclear to what extent the outcomes of renal denervation in CKD are related to direct effects of lowering sympathetic drive or indirect effects of BP reduction due to RDN [75].

This uncertainly arises partly from limited data on the sympathetic effects of renal denervation in CKD. In a proof-of-concept study, conducted on nine ESKD patients undergoing hemodialysis, authors specifically evaluated the impact of endovascular RDN on muscle sympathetic nerve traffic [76]. The results demonstrated a reduction of approximately 20% in the sympathoinhibitor effects of RDN up to a 12-month follow-up period.

## Effect of Renal Denervation

### Trials

Symplicity HTN-1, symplicity HTN-2, and other smaller trials presented intriguing results concerning the potential role of RDN in treating RH within the hypertensive population. However, the symplicity HTN-3 trial yielded less encouraging outcomes. While it confirmed the safety of the RDN, it failed to prove its efficacy in comparison to a sham procedure for BP control [77, 78].

In contrast, data from registries have indicated that RDN could effectively reduce BP even in patients with renal failure. Indeed, Ott. et al., analyzing 3-year follow-up data from the Global Symplicity Registry, found that the use of RDN resulted in similar reductions of office and ABPM-24 h in both 475 patients with eGFR < 60 mL/min/1.73 m^2^ and 1505 patients with eGFR ≥ 60 mL/min/1.73 m^2^ [79].

Despite our incomplete understanding of the true efficacy of RDN, the recent 2023 ESH guidelines [9], with a grade of evidence IIB, consider RDN a valuable additional treatment option for RH patients, but only when eGFR is > 40 mL/min/1.73 m^2^. However, it is worth noting that clinical trials have thus far excluded patients with eGFR < 45 mL/min/1.73 m2 in their enrollment.

### Other Studies

Over the past decade, encouraging data on RDN have emerged from pilot studies characterized by small sample sizes, within study populations with ESKD and RH (Table 1) [80•]. The first report into the ablation of renal sympathetic nerve using a radiofrequency catheter was documented in a 39-year-old patient undergoing dialysis. In this innovative case report, Di Daniele et al. observed a progressive reduction in both systolic and diastolic BP, from 180 ± 15 and 105 ± 11 mmHg at baseline to 155 ± 14 and 90 ± 10 mmHg at 1 month, respectively [81].

**Table 1:** Main characteristics of studies assessing the impact of renal denervation in patients on dialysis with resistant hypertension

**Author,**	**Country**	**Sample**		**Study Design**	**Follow-up**	**Results**
**year [ref]**		***N***	**Characteristics**		**Months**	
Schlaich el al., 2013 [82]	Germany	9	HD	Single center Prospective Uncontrolled	12	Mean 24H ABPM reduction: 19 mmHg (SBP) 7 mmHg (DBP)
Hoye et al., 2017 [81]	New Zealand	9	HD/PD	Single center Safety Proof-of-concept	12	Mean 24H ABPM reduction: 24 mmHg (SBP) 13 mmHg (DBP)
Ott et al., 2019 [83]	Germany	6	HD	Single center Case-series	6	Mean 24H ABPM reduction: 20 mmHg (SBP) 15 mmHg (DBP)
Scalise et al., 2020 [85•]	Italy	12	HD	Single center Prospective Controlled	12	Mean 24H ABPM reduction: 26 mmHg (SBP) 14 mmHg (DBP)

*24H ABPM* 24-h ambulatory blood pressure monitoring, *DBP* diastolic blood pressure, *HD* hemodialysis, *PD* peritoneal dialysis, *SBP* systolic blood pressure

Subsequently, this initial data was confirmed by Schlaich et al. [82] which illustrated the efficacy of RDN in twelve patients with HD and RH. Notably, office systolic BP significantly decreased from 166 ± 16.0 to 148 ± 11, 150 ± 14, and 138 ± 17 mmHg at 3, 6, and 12 months post-RDN, respectively. It is worth noting that the application of RDN was limited in three patients due to the presence atrophic renal arteries.

In contrast, Ott et al. achieved noteworthy results in a 29-year-old HD woman who had RH with small native renal arteries (< 4 mm). Through catheter-based renal denervation performed on both native kidneys, notable systolic (38 mmHg) and diastolic BP (30 mmhg) reduction were obtained.

The feasibility and safety of bilateral RDN in HD patients with small renal arteries were confirmed through a case series in which systolic and diastolic ABPM-24 h were significantly reduced an average of 20 ± 17 and 15 ± 12 mmHg (*p* = 0.043) at 6 months following the procedure, both for daytime and nighttime values. Notably, it is intriguing to point out that even though three out of six patients had renal arteries with a diameter less than 4 mm, no procedural adverse events were recorded [83].

These findings were consistent with another case series by Pietilä-Effati et al., involving four HD patients with RH (with a mean ABPM-24 h of 175/95 mmhg) treated with intravascular renal denervation at a single center. Similarly, in this study, no procedure-related adverse events were observed. Remarkably, in the 50% of cases, antihypertensive therapy was discontinued, and one patient maintained normotension for an impressive 24-month duration. [84].

The enduring efficacy of RDN in reducing the BP was further corroborated by Scalise et al., [85•] in a study including 24 log-term ESKD patients (mean 55 ± 16 years of dialysis) with RH treated, who were already on multiple antihypertensive medications. This reduction in BP persisted throughout the 1-year follow-up period, as evidenced through ambulatory and office BP measurements.

Furthermore, Hoye et al. [81] unveiled further significant effects of RDN treatment in a study involving nine patients with ESKD, of which six undergoing hemodialysis and three on peritoneal dialysis. Beyond the impact on BP, reductions in left ventricular mass index (LVMI) were highlighted. Impressively, these effects became evident as early as 3 months following the procedure and persisted for up to 12 months.

Collectively, to date, a unique meta-analysis has been conducted, focusing on impact of RDN in 238 RH patients with CKD. This analysis encompassed not only patients undergoing HD but also those with CKD stages 1–5, drawing data from 11 single-center studies, non-randomized, uncontrolled [86]. The results revealed that RDN exhibited effectiveness in reducing both office BP and 24-h ambulatory BP, from baseline to 1 month (*p* < 0.05) and notable reductions in 24-h ambulatory systolic (*p* < 0.001) and diastolic (*p* = 0.001) BP over a span of at least 24 months.

### Case Report

**Fig. 1:**
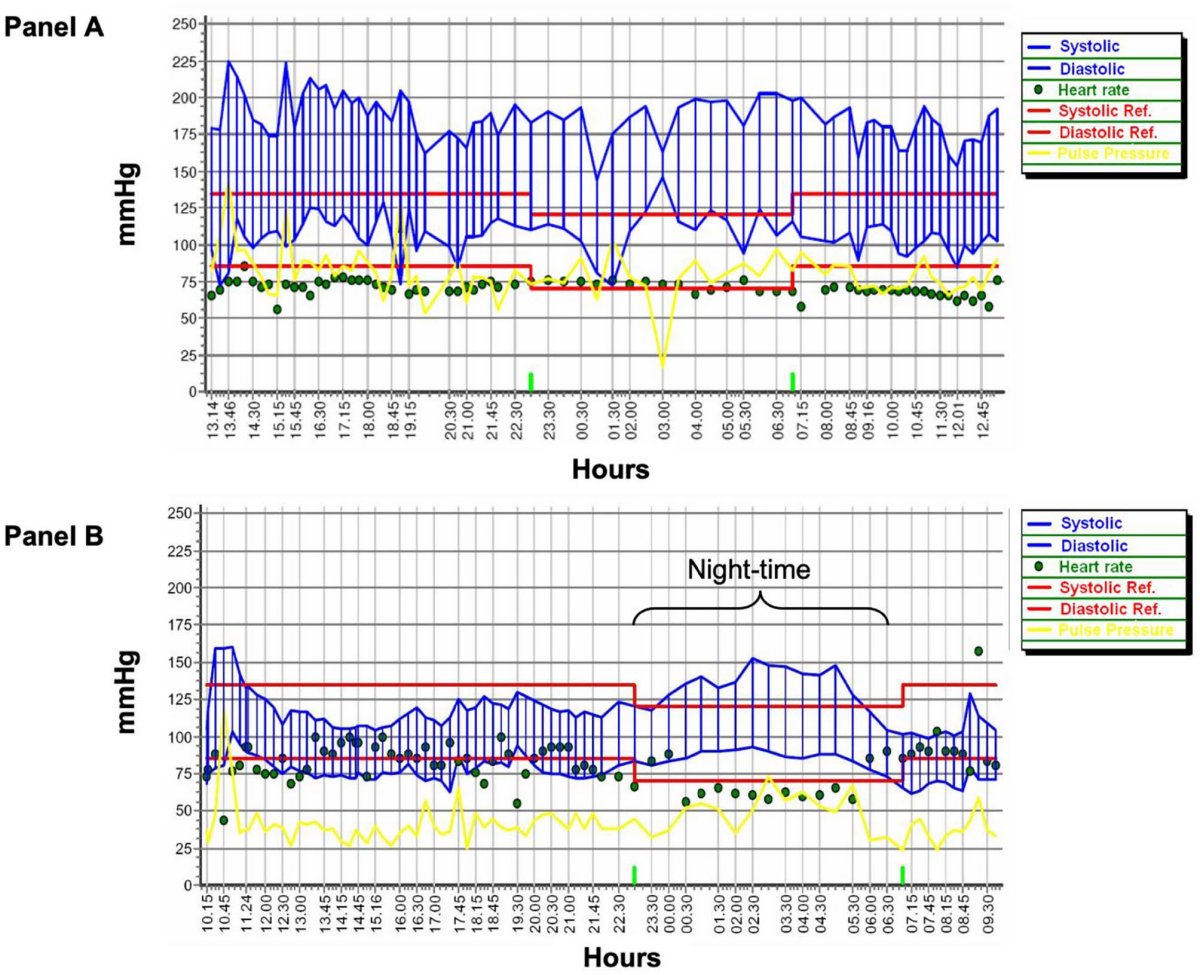
Twenty-four-hour ambulatory blood pressure measurement of patient. **A** Twenty-four-hour ambulatory blood pressure measurement before renal denervation procedure, showing an uncontrolled hypertension despite anti-hypertensive treatment up to 10 medications. **B** Twenty-four-hour ambulatory blood pressure measurement, 48-h after the renal denervation procedure, showing a better (BP) control during daytime, while remains a reverse dipper profile in the nighttime BP values

A 47-year-old male, with a long history of HT and renal amyloidosis since 2009, has been following a dialysis program for the past 2 years, three times weekly. His antihypertensive therapy consisted of nifedipine 60 mg twice daily, ramipril 5 mg twice daily, furosemide 500 mg daily, doxazozine 4 mg three time daily, and valsartan 320 mg daily. In the last year, he was admitted to the emergency department three times in 2 months for hypertensive crisis (averaged BP of 250/140 mmHg) and acute encephalopathy with severe headache, vomiting, and blurred vision. Ocular fundus examination showed a grade III retinopathy. Brain CT showed no signs of bleeding, while electrocardiogram revealed sinus bradycardia with left ventricular hypertrophy, left anterior hemi-block, and left axial deviation, without alterations of the S/T segment. Echocardiography documented hypertensive heart disease with a LVMI of 165.3 g/m2; left ventricular ejection fraction (64%) and contractility function were normal. Laboratory tests showed hyperkalemia (5.34 mmol/L). The diagnosis of RH was confirmed by the 24-h ambulatory BP monitoring (Fig. 1, panel A) and adherence to anti-hypertensive treatment was assessed by liquid chromatography–mass spectrometry analysis for drug metabolites in urine. After a multidisciplinary discussion, the patients underwent RDN using a third generation SpyralTM catheter, a device with multi-electrode configuration associated with reduced procedural time, contrast use, and radiation exposure. The written consent of patients was collected.

The RDN procedure was completed without complications, and after 48 h, systolic BP decreased by up to 40 mmHg (Fig. 1, panel B) and was controlled with four antihypertensive drugs 1 month later. After 6 months, office BP remained controlled at 130/80 mmHg and LVMI significantly reduced from baseline (165.3 vs. 149.4 g/m2, *p* < 0.001).

To the best of our best knowledge, we report the first case report of RDN performed in amyloidotic patients on hemodialysis. In clinical practice, our case reveals that RDN could be technically feasible and potentially effective in ESKD patients’ dialysis dependent. While current evidence does not establish that RDN is superior to intensive (optimal) anti-hypertensive drug therapy in improving cardiac remodeling and function, our experience showed that RDN improved the quality of life of our patient and reduced the daily intake of anti-hypertensive medications from ten to four pills. Moreover, the significant improvements in office and ambulatory BP as well as LVMI values, observed in our patients, might carry significant clinical implications in reducing the very-high global cardiovascular risk faced by hypertensive patients on dialysis.

## Conclusion

Endovascular RDN can induce a sympatholytic effect, resulting in a significant reduction in BP and left ventricular mass index values. This procedure should be considered in carefully selected cases, particularly in young adults with dialysis-dependent ESKD and true RH, in order to mitigate the risk of severe, life-threatening organ damage. However, further larger-scale, randomized and sham-controlled clinical trials should be planned and conducted specifically in ESKD patients on dialysis treatment with RH.

## Data Availability

The original contributions presented in the study are included in the article, further inquiries can be directed to the corresponding author.
