# Correction to: Does Renal Denervation a Reasonable Treatment Option in Hemodialysis-Dependent Patient with Resistant Hypertension? A Narrative Review

**DOI:** 10.1007/s11906-023-01270-4

**Published:** 2023-10-10

**Authors:** Alberto Mazza, Fabio Dell’Avvocata, Gioia Torin, Francesca Bulighin, Yuri Battaglia, Fulvio Fiorini

**Affiliations:** 1 ESH Excellence Hpertension Centre and Dept. of Internal Medicine, Santa Maria della Misericordia General Hospital, AULSS 5 Polesana, Rovigo, Italy; 2 Cardiovascular Diagnosis and Endoluminal Interventions Unit, Santa Maria della Misericordia General Hospital, AULSS 5 Polesana, Rovigo, Italy; 3 Department of Medicine, University of Verona, 37129 Verona, Italy; 4 Nephrology and Dialysis Unit, Pederzoli Hospital, Via Monte Baldo, 24, 37019 Peschiera del Garda, Italy; 5 Nephrology, Dialysis and Dietology Unit, Santa Maria della Misericordia General Hospital, AULSS 5 Polesana, Rovigo, Italy

**Correction to: Current Hypertension Reports** 10.1007/s11906-023-01264-2

The article “Does Renal Denervation a Reasonable Treatment Option in Hemodialysis-Dependent Patient with Resistant Hypertension? A Narrative Review”, was originally published electronically on the publisher’s internet portal on 6 September 2023 with error on the tagging of the author names (first names and last names were interchanged) and affiliation of the third author.

The correct tagging of the author names are as follows:

- Author 1
  - First Name: Alberto
    Last Name: Mazza
- Author 2
  - First Name: Fabio
    Last Name: Dell'Avvocata
- Author 3
  - First Name: Gioia
    Last Name: Torin
- Author 4
  - First Name: Francesca
    Last Name: Bulighin
- Author 5
  - First Name: Yuri
    Last Name: Battaglia
- Author 6
  - First Name: Fulvio
    Last Name: Fiorini

Gioia Torin (third author) should be affiliated to ESH Excellence Hpertension Centre and Dept. of Internal Medicine, Santa Maria della Misericordia General Hospital, AULSS 5 Polesana, Rovigo, Italy.

The original article has been corrected.

